# Association between metabolic obesity phenotypes and the risk of developing prostate cancer: a propensity score matching study based on Xinjiang

**DOI:** 10.3389/fendo.2024.1442740

**Published:** 2024-08-06

**Authors:** Jinru Wang, Aireti Apizi, Hao Qiu, Ning Tao, Hengqing An

**Affiliations:** ^1^ School of Public Health, Xinjiang Medical University, Urumqi, China; ^2^ Department of Urology, The First Affiliated Hospital of Xinjiang Medical University, Urumqi, China

**Keywords:** prostate cancer, metabolic obesity phenotypes, propensity score matching, metabolic risk factors, conditional logistic regression

## Abstract

**Background:**

Obesity-induced metabolic dysfunction increases the risk of developing tumors, however, the relationship between metabolic obesity phenotypes and prostate cancer (PCa) remains unclear.

**Methods:**

The term metabolic obesity phenotypes was introduced based on metabolic status and BMI categories. Participants were categorized into four groups: metabolically healthy nonobesity (MHNO), metabolically healthy obesity (MHO), metabolically unhealthy nonobesity (MUNO), and metabolically unhealthy obesity (MUO). Propensity score matching was conducted based on age, ethnicity, marriage, etc. Univariate and multivariate conditional logistic regression analyses were used to assess the relationship between metabolic obesity phenotypes, metabolic risk factors, and PCa. Sensitivity analysis was performed to verify the robustness of the results.

**Results:**

After propensity score matching among 564 PCa patients and 1418 healthy individuals, 209 were selected for each of the case and control groups. There were no statistically significant differences in the basic characteristics between the two groups. Univariate and multivariate conditional logistic regression suggested that the risk of developing PCa in both MHO and MUO individuals was higher than in MHNO individuals. Specifically, the risk of developing PCa in MHO individuals was 2.166 times higher than in MHNO individuals (OR=2.166, 95%CI: 1.133-4.139), and the risk in MUO individuals was is 2.398 times higher than in MHNO individuals(OR=2.398, 95%CI:1.271-4.523). Individuals with hyperglycemia and elevated triglycerides also had a higher risk of developing PCa (hyperglycemia:OR=1.488, 95%CI: 1.001-2.210; elevated triglycerides: OR=2.292, 95%CI: 1.419-3.702). Those with more than or equal to three metabolic risk factors had an increased risk of PCa (OR=1.990, 95%CI: 1.166-3.396). Sensitivity analysis indicated an increased risk of PCa in MUO individuals compared to MHNO individuals.

**Conclusion:**

In this retrospective study, individuals with MHO and MUO had a higher risk of developing PCa.

## Introduction

PCa is a complex, hormone-dependent disease and the most common malignant cancer in males (1). The development of PCa involves multiple factors, with recognized risk factors including age, smoking, family history, and genetic susceptibility/genetic variants (2, 3), a minority of patients may have both granulomatous prostatitis and PCa (4). Additionally, exogenous factors such as obesity and metabolic syndrome have been associated with the development and progression of PCa, as well as with an increased risk of adverse pathology (5). Obesity and overweight are growing public health problems driven by unhealthy eating patterns and lifestyles. Since 1980, approximately one-third of the global population has been classified as overweight or obesity (6).

Obesity is strongly linked to numerous metabolic abnormalities, including hyperinsulinemia, elevated free fatty acid levels, high triglycerides, and reduced high density lipoprotein cholesterol. The metabolism related thyroid hormones have an effect on both the tumour and its microenvironment, and involve in tumour growth, invasion and metastasis (7). The World Obesity Federation predicts that the prevalence of obesity will increase 24% by 2035, with nearly 2 billion people expected to suffer from metabolic disorders (8). As a metabolic pathogenic status, obesity is often accompanied by systemic chronic low-grade inflammation and elevated levels of immune markers such as leptin, IL-6, and TNF, which may influence tumor growth (9, 10). Metabolic dysfunction due to obesity increases cancer risk (11). PCa is considered one of the cancers potentially linked causally to obesity. While many studies have reported a correlation between obesity and PCa risk, the results have been conflicting (12–14). To resolve these conflicting findings, further investigation into the underlying mechanisms is necessary.

Obesity is increasingly considered a heterogeneous disease (15). There is evidence that some individuals with a BMI in the overweight or obese range do not exhibit metabolic disorders, maintaining normal blood glucose levels, insulin sensitivity, and low blood pressure (16, 17). Conversely, a significant proportion of individuals with a normal weight also present metabolic abnormalities (18). Metabolic dysfunction is recognized as a key risk factor for obesity-related cancers, regardless of the presence of obesity (11). Body mass index (BMI), as a solitary measure, does not distinguish between fat and muscle mass, and does not account for the increased risk associated with variations in body fat distribution. In contrast, metabolic health provides a comprehensive perspective on the body’s metabolic processes and overall physical health. Sims first introduced the concept of a metabolically healthy obesity (MHO) phenotype in 2001 (19). This concept integrates metabolic health and obesity, defining individuals with both metabolic unhealth and obesity as having metabolically unhealthy obesity (MUO) (20). Some studies have indicated that MHO individuals have a reduced cancer risk compared to MUO individuals, suggesting that an unhealthy metabolism may play a critical role in cancer development (21).

Previous literature has aimed to clarify the relationship between metabolic syndrome and PCa risk, identifying metabolic syndrome as a complex clinical feature characterized by obesity (22–24). While obesity is generally associated with a higher risk of most cancer types, a recent mendelian randomization study suggested that obesity might reduce PCa risk (25). Currently, the relationship between obesity, metabolic status, and PCa risk remains controversial. Thus, in this study, we evaluated the correlation between metabolic obesity phenotypes and PCa, to investigate whether these phenotypes influence the risk of developing PCa. The aim of this study was to evaluate the relationship between metabolic obesity phenotypes and PCa based on data from a large-scale general hospital in Xinjiang, and to identify the risk factors of developing PCa.

## Methods

### Data source

**Figure 1:**
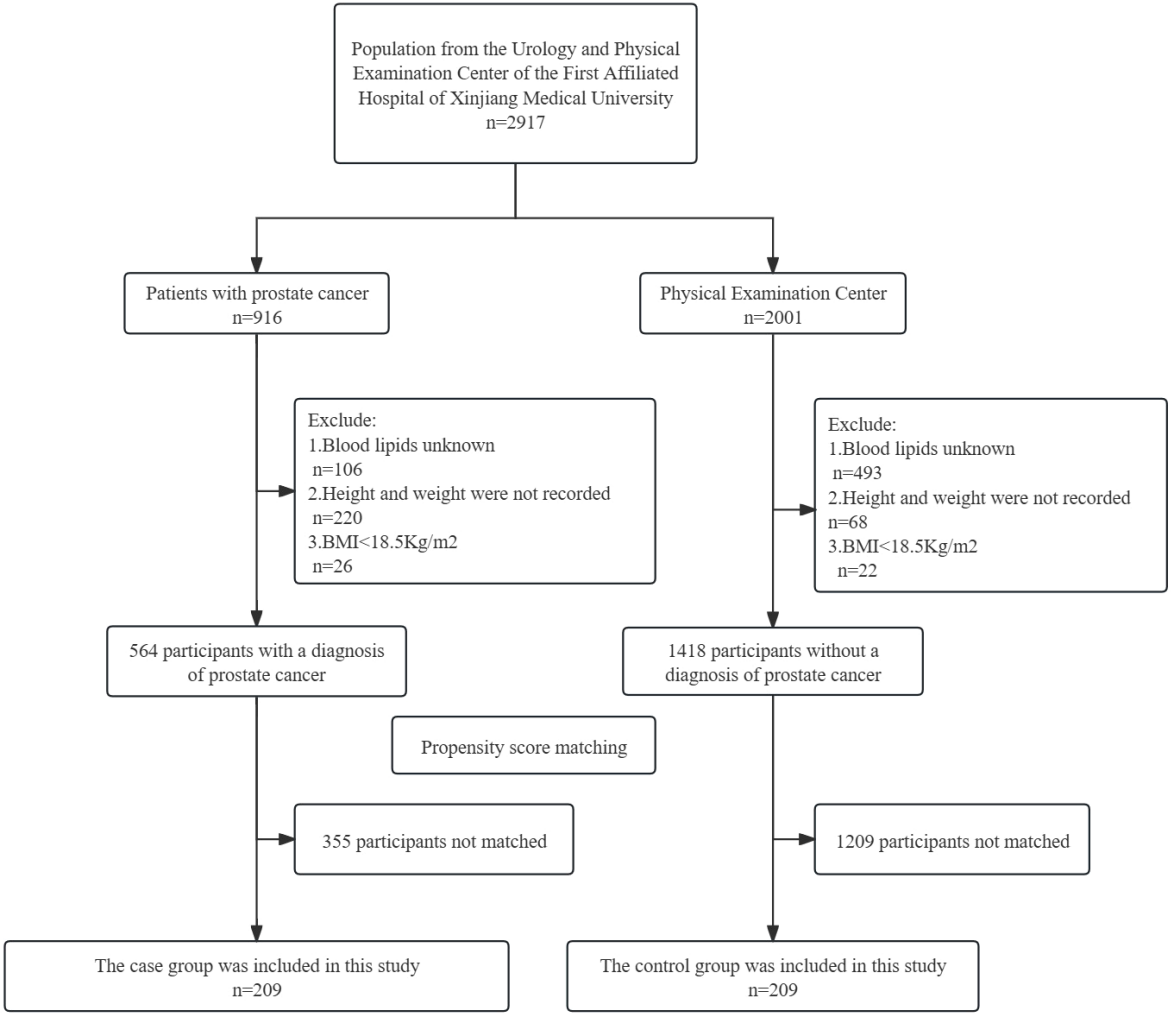
Flowchart of inclusion and exclusion of study participants.

We studied 2917 subjects from the Department of Urology of the First Affiliated Hospital of Xinjiang Medical University from 2020 to 2023. A total of 1982 participants remained for analysis after the exclusion. This study included 564 patients with PCa confirmed by prostate biopsy and puncture, and 1,418 people who underwent physical examination at the physical examination center. After propensity score matching, 209 were selected for each of the case and control groups; **Figure 1**.

Inclusion criteria for control group: (1) males attending for the physical examination at the Physical Examination Centre of the First Affiliated Hospital of Xinjiang Medical University during the same period; (2) complete physical examination data; (3) can read, understand and sign the informed consent form.

Exclusion criteria for control group: (1) participants with any type of cancer or history of cancer; (2) participants with BMI <18.5Kg/m^2^.

Inclusion criteria for case group: (1) patients first diagnosed with PCa by prostate biopsy from 2020 to 2023; (2) complete test data; (3) can read, understand and sign the informed consent form.

Exclusion criteria for case group: (1) PCa patients with a history of other types of cancer; (2) patients with BMI <18.5Kg/m^2^.

The protocol was approved by the Ethics Committee of the First Affiliated Hospital of the Xinjiang Medical University (Approval No. 20220 308-166), and all study participants signed an informed consent form with a clear understanding of the purpose of the study protocol.

### Data collection and measurements

A physical examination was conducted to obtain anthropometric characteristics (height and weight), blood pressure, and blood sample data. Participants were asked to wear light clothing and have their height and weight measured while barefoot. Blood samples were collected early in the morning after fasting for 10-12 hours, using a vein in front of the elbow. These samples were analyzed for fasting fasting blood glucose, total cholesterol, triglycerides, high density lipoprotein cholesterol, low density lipoprotein cholesterol, creatinine, and uric acid. Laboratory technicians enzymatically analyzed all blood samples using an automated analyzer, with fasting blood glucose, triglycerides, and high density lipoprotein cholesterol measured by hexokinase, enzyme, and clearance methods, respectively.

### Definition of the metabolic obesity phenotypes

According to the Adult Treatment Panel III (ATP-III) criteria, the International Diabetes Federation (IDF) consensus, and the JACC Health Promotion Series (26–28), we defined metabolic risk factors: (1) Elevated triglycerides (blood triglyceride≥1.7 mmol/L or 150 mg/dL). (2) Hypertension (systolic pressure≥130 mmHg or diastolic pressure≥85 mmHg). (3) Hyperglycemia (fasting blood glucose ≥ 5.6 mmol/L or 100mg/dL). (4) Reduced HDL-c (HDL-c <1.04 mmol/L or 40mg/dL). BMI (Kg/m^2^) is calculated based on weight (Kg) divided by the square of height (m). The study population was categorized according to the WHO criteria for Asian populations, BMI threshold is 25 Kg/m^2^, nonobesity defined as BMI<25Kg/m^2^ (normal weight: 18.5Kg/m^2^ ≤ BMI<23Kg/m^2^, overweight: 23Kg/m^2^ ≤ BMI<25Kg/m^2^), individuals with a BMI ≥ 25 Kg/m^2^ were classified as obesity (29, 30). Evidence suggests that there is a collinearity between waist circumference and BMI (31), therefore diagnostic criteria for metabolic status did not include waist circumference. Metabolically unhealthy status is defined as having two or more of the above metabolic risk factors. Subjects were categorized into four metabolic obesity phenotypes based on the combination of metabolic status and BMI: (1) Metabolically healthy nonobesity (MHNO): No or only one metabolic risk factor and BMI <25kg/m^2^. (2) Metabolically healthy obesity (MHO): No or only one metabolic risk factor and BMI ≥ 25 kg/m^2^. (3) Metabolically unhealthy nonobesity (MUNO): two or more metabolic risk factors and BMI<25kg/m^2^. (4) Metabolically unhealthy obesity (MUO): two or more metabolic risk factors and BMI ≥ 25 kg/m^2^.

### Statistical analysis

PASS 15 software was used to calculate the sample size required for this study. For missing secondary study variables, we implemented multiple interpolation. Baseline information before propensity score matching were compared using t tests, Mann-Whitney U test, and χ^2^ test. Nearest neighbor matching propensity score matching in a 1:1 ratio for case and control groups, caliper value of 0.05, to reduce the interference of confounding factors. Baseline information after propensity score matching were compared using paired t-test, paired rank sum test, and paired χ^2^ test. Association between metabolic obesity phenotypes, number of metabolic risk factors and the risk of developing PCa were assessed by univariate and multivariate conditional logistic regression. Sensitivity analysis to ensure the robustness of the results. All statistical analyses were conducted using SPSS and R (version 4.2.0). All statistical tests were 2-sided, with *P* < 0.05 considered statistically significant.

## Results

### Baseline characteristics

Before propensity score matching, there were 564 PCa patients in the case group and 1,418 participants in the control group who participated in the physical examination. The median age of the case and control groups were 72 and 44 years, respectively. The metabolic obesity phenotypes were statistically different between two groups (*P* < 0.05); **Table 1**.

**Table 1:** Baseline characteristics before propensity score matching.

Parameters	Prostate cancer (n=564)	Non-prostate cancer (n=1418)	χ^2^/Z	*P* value
Age/[P_50_(P_25_,P_75_), (years)]	72.00 (66.00, 78.00)	44.00 (35.00, 51.00)	-30.902	<0.001
Ethnicity/[case(%)]			7.152	0.028
The Han nationality	371 (65.78%)	1019 (71.86%)		
Uyghur ethnic group	115 (20.39%)	235 (16.57%)		
Other	78 (13.83%)	164 (11.57%)		
Marital status/[case(%)]			31.931	<0.001
Married	529 (93.79%)	1196 (84.34%)		
Unmarried, divorced, widowed	35 (6.21%)	222 (15.66%)		
Blood potassium/[P_50_(P_25_,P_75_), mmol/L]	3.80 (3.58, 4.12)	4.08 (3.85, 4.33)	-12.012	<0.001
Blood sodium/[P_50_(P_25_,P_75_), mmol/L]	139.90 (137.56, 142.31)	141.76 (140.00, 143.20)	-9.962	<0.001
Blood chloride/[P_50_(P_25_,P_75_), mmol/L]	104.80 (102.90, 107.40)	103.70 (101.70, 105.50)	-8.275	<0.001
Blood Calcium/[P_50_(P_25_,P_75_), mmol/L]	2.23(2.14, 2.32)	2.33 (2.27, 2.40)	-15.671	<0.001
BMI/[P_50_(P_25_,P_75_), Kg/m^2^]	24.61(22.00, 27.00)	24.69 (22.76, 26.64)	-0.787	0.431
Fasting blood glucose (FBG)/[P_50_(P_25_, P_75_), mmol/L]	5.49(4.79, 6.84)	5.04(4.68, 5.50)	-8.694	<0.001
Blood triglyceride/[P_50_(P_25_, P_75_), mg/dL]	126.26 (92.14, 171.00)	108.98 (80.85, 143.53)	-7.004	<0.001
Total cholesterol/[P_50_(P_25_, P_75_), mmol/L]	4.13(3.49, 4.79)	4.78 (4.19, 5.38)	-13.119	<0.001
High density lipoprotein cholesterol (HDL-c)/[P_50_(P_25_, P_75_), mg/dL]	39.44(32.48, 47.95)	44.08 (38.28, 50.66)	-8.541	<0.001
Low density lipoprotein cholesterol (LDL-c)/[P_50_(P_25_, P_75_), mmol/L]	2.61(2.07, 3.20)	2.93 (2.47, 3.46)	-7.735	<0.001
Uric acid/[P_50_(P_25_, P_75_),μmol/L]	316.95 (255.62, 370.98)	324.40 (277.25, 368.75)	-2.543	0.011
Albumin/[P_50_(P_25_, P_75_), g/L]	68.80 (63.60, 73.32)	46.80(44.70, 49.10)	-28.602	<0.001
Globulin/[P_50_(P_25_, P_75_), g/L]	39.16(35.80, 42.30)	27.10(24.50, 30.60)	-27.395	<0.001
γ-Glutamyl Transferase (GGT)/[P_50_(P_25_, P_75_),μ/L]	22.05 (17.00, 32.23)	25.61(18.38, 37.30)	-4.523	<0.001
Alkaline phosphatase/[P_50_(P_25_, P_75_), U/L]	73.00(60.00, 97.27)	72.01(60.93, 85.58)	-2.763	0.006
Metabolic obesity phenotypes/[case(%)]			148.226	<0.001
Metabolically healthy nonobesity (MHNO)	145 (25.71%)	598 (42.17%)		
Metabolically healthy obesity (MHO)	98 (17.38%)	421 (29.69%)		
Metabolically unhealthy nonobesity (MUNO)	152 (26.95%)	163 (11.50%)		
Metabolically unhealthy obesity (MUO)	169 (29.97%)	236 (16.64%)		

We calculated that the required sample size after propensity score matching on both the case and control groups was 175 (32, 33). After propensity score matching, 209 PCa patients and 209 non-PCa participants were included in the case and control groups. The mean age of the case and control groups was 67.19 and 67.05. There were no statistically significant differences between the two groups in the basic characteristics of age, race and marital status (*P* > 0.05). The metabolic obesity phenotypes were statistically different between two groups (*P* < 0.05); **Table 2**.

**Table 2:** Baseline characteristics after propensity score matching.

Parameters	Prostate cancer (n=209)	Non-prostate cancer (n=209)	χ^2^/t/Z	*P* value
Age/[ $\bar{\chi }$ ±s, (years)]	67.19 ± 0.62	67.05 ± 0.61	0.538	0.591
Ethnicity/[case(%)]			2.875	0.238
The Han nationality	131 (62.68%)	142 (67.94%)		
Uyghur ethnic group	50 (23.92%)	36 (17.22%)		
Other	28 (13.40%)	31 (14.83%)		
Marital status/[case(%)]			0.622	0.430
Married	200 (95.69%)	203 (97.13%)		
Unmarried, divorced, widowed	9 (4.31%)	6 (2.87%)		
Blood potassium/[P_50_(P_25_,P_75_), mmol/L]	3.79 (3.53, 4.01)	3.82 (3.61, 4.11)	-1.441	0.149
Blood sodium/[P_50_(P_25_,P_75_), mmol/L]	140.00 (137.71, 142.28)	140.46 (138.39, 142.50)	-1.113	0.266
Blood chloride/[P_50_(P_25_,P_75_), mmol/L]	104.50 (102.80, 107.10)	105.00 (102.90, 107.00)	-0.692	0.489
Blood Calcium/[P_50_(P_25_,P_75_), mmol/L]	2.25 (2.15, 2.33)	2.27 (2.17, 2.35)	-1.863	0.062
BMI/[P_50_(P_25_,P_75_), Kg/m^2^]	24.98(22.00,27.00)	24.00(21.82,26.00)	2.874	0.004
fasting blood glucose (FBG)/[P_50_(P_25_, P_75_), mmol/L]	5.45(4.74,6.54)	5.19(4.61,6.22)	0.994	0.320
Blood triglyceride[P_50_(P_25_, P_75_), mg/dL]	127.58(89.49,171.44)	107.21(84.17,141.32)	5.029	<0.001
Total cholesterol/[P_50_(P_25_, P_75_), mmol/L]	4.28(3.55,4.90)	4.22(3.62,4.81)	0.716	0.474
High density lipoprotein cholesterol (HDL-c)/[P_50_(P_25_, P_75_), mg/dL]	37.51(32.29,47.56)	39.44(32.48,47.56)	0.362	0.717
Low density lipoprotein cholesterol (LDL-c)/[P_50_(P_25_, P_75_), mmol/L]	2.74(2.19,3.25)	2.59(2.10,3.17)	2.011	0.044
Uric acid/[P_50_(P_25_, P_75_),μmol/L]	314.00(250.95,377.90)	305.00(251.73,358.69)	0.856	0.392
Albumin/[P_50_(P_25_, P_75_), g/L]	68.27(64.05,73.33)	67.07(60.33,72.59)	2.512	0.012
Globulin/[P_50_(P_25_, P_75_), g/L]	39.70(35.84,42.80)	38.90(32.94,41.80)	2.611	0.009
γ-Glutamyl Transferase (GGT)/ [P_50_(P_25_, P_75_),μ/L]	23.00(17.49,32.50)	23.00(17.00,34.15)	-0.286	0.775
Alkaline phosphatase/[P_50_(P_25_, P_75_), U/L]	74.00(58.65,97.10)	74.00(61.49,90.67)	0.709	0.478
Metabolic obesity phenotypes/[case(%)]			8.059	0.010
Metabolically healthy nonobesity (MHNO)	50(23.92%)	74(35.41%)		
Metabolically healthy obesity (MHO)	49(23.44%)	44(21.05%)		
Metabolically unhealthy nonobesity (MUNO)	55(26.32%)	53(25.36%)		
Metabolically unhealthy obesity (MUO)	55(26.32%)	38(18.18%)		

### Univariate and multivariate analyses of metabolic obesity phenotypes and PCa

After propensity score matching, univariate conditional logistic regression analysis showed that the risk of developing PCa in both MHO and MUO individuals was higher than in MHNO individuals (MHO: OR=1.855,95%CI: 1.007-3.417; MUO: OR=2.310,95%CI: 1.289-4.140). Albumin, globulin and alkaline phosphatase were positively associated with the risk of developing PCa; **Table 3**.

**Table 3:** Impact of metabolic obesity phenotypes on the risk of developing PCa after propensity score matching.

Parameters	Univariate conditional logistic regression analysis		Multivariate conditional logistic regression analysis	
	OR(95%CI)	*P* value	OR(95%CI)	*P* value
Metabolic obesity phenotypes		0.037		0.032
Metabolically healthy nonobesity (MHNO)	1.000(ref.)		1.000(ref.)	
Metabolically healthy obesity (MHO)	1.855(1.007-3.417)	0.047	2.166(1.133-4.139)	0.019
Metabolically unhealthy nonobesity (MUNO)	1.707(0.957-3.046)	0.070	1.574(0.841-2.947)	0.156
Metabolically unhealthy obesity (MUO)	2.310(1.289-4.140)	0.005	2.398(1.271-4.523)	0.007
Uric acid(μmol/L)	1.001(0.999-1.003)	0.368	—	**—**
Total cholesterol (mmol/L)	1.036(0.839,1.280)	0.740	—	**—**
Low density lipoprotein cholesterol (LDL-c) (mmol/L)	1.263(0.989-1.614)	0.061	—	**—**
Albumin (g/L)	1.034(1.012-1.056)	0.002	1.003(0.965-1.043)	0.868
Globulin(g/L)	1.051(1.016-1.087)	0.004	1.072(1.006-1.143)	0.033
γ-Glutamyl Transferase (GGT) (μ/L)	0.998(0.990-1.005)	0.530	—	**—**
Alkaline phosphatase(U/L)	1.006(1.002-1.011)	0.006	1.009(1.004-1.015)	0.001

The factors included in the univariate conditional logistic regression that were statistically significant (*P* < 0.05) were followed by multivariate conditional logistic regression. The results demonstrated that the risk of developing PCa was 2.166 times higher in MHO individuals than in MHNO individuals (OR=2.166, 95%CI: 1.133-4.139), and the risk in MUO individuals was 2.398 times higher than in MHNO individuals (OR=2.398, 95%CI: 1.271-4.523). Globulin and alkaline phosphatase were risk factors of developing PCa. Specifically, there was a 7.2% increase in PCa risk for every 1-unit rise in globulin (OR=1.072, 95%CI: 1.006-1.143). Additionally, for every 1-unit increase in alkaline phosphatase, there was a 0.9% increase in the risk of developing PCa (OR=1.009, 95%CI: 1.004-1.015); **Table 3**.

### Association between metabolic risk factors and PCa

#### Relationship between metabolic risk factors and PCa

As mentioned above, we defined metabolic risk factors as hypertension, hyperglycemia, elevated triglycerides, and reduced HDL-c. The results indicated that hyperglycemia and elevated triglycerides were risk factors of PCa. The risk of developing PCa in individuals with hyperglycemia was 1.488 times higher than in individuals with non-hyperglycemia (OR=1.488, 95%CI: 1.001-2.210). Additionally, the risk of developing PCa in individuals with elevated triglycerides was 2.292 times higher than in individuals with non-elevated triglycerides (OR=2.292, 95%CI: 1.419-3.702). There were no statistically significant differences between other metabolic risk factors and PCa (*P*>0.05); **Table 4**.

**Table 4:** Metabolic risk factors and risk of developing PCa.

Metabolic risk factors	OR(95%CI)	*P* value
Hypertension		
no	1.000(ref.)	
yes	0.940(0.631-1.400)	0.761
Hyperglycemia		
no	1.000(ref.)	
yes	1.488(1.001-2.210)	0.049
Elevated triglycerides		
no	1.000(ref.)	
yes	2.292(1.419-3.702)	0.001
Reduced HDL-c		
no	1.000(ref.)	
yes	1.140(0.780-1.667)	0.499

#### Relationship between the number of metabolic risk factors and PCa

The risk of developing PCa individuals with more than or equal to three metabolic risk factors was 1.990 times higher than those with less than or equal to one metabolic risk factor (OR=1.990, 95%CI: 1.166-3.396). With the number of metabolic risk factors increasing, the risk of developing PCa also increases; **Table 5**.

**Table 5:** Relationship between the number of metabolic risk factors and the risk of developing PCa.

Number of metabolic risk factors	OR(95%CI)	*P* value
		0.041
≤1	1.000(ref.)	
2	1.216(0.770-1.921)	0.401
≥3	1.990(1.166-3.396)	0.012

### Sensitivity analyses by adjusted study population

To demonstrate the robustness of the results, we performed sensitivity analyses. The results of the sensitivity analyses showed that our results were stable in the various populations.

### Redefining obesity

We use different criteria to define obesity, BMI classification according to the Chinese Obesity Working Group (34). BMI≥28Kg/m^2^ was considered obesity. Re-analysis of the relationship between metabolic obesity phenotypes and the risk of developing PCa, to ensure robustness of results. The results showed that the risk of developing PCa was 4.598 times higher in MHO individuals than in MHNO individuals (OR= 4.598, 95%CI: 1.476 -14.324), and the risk in MUO individuals was 3.296 times higher than in MHNO individuals (OR=3.296, 95%CI: 1.357 -8.007); **Table 6**.

**Table 6:** Definition of obesity by BMI≥28Kg/m^2^.

Parameters	Univariate conditional logistic regression analysis		Multivariate conditional logistic regression analysis	
	OR(95%CI)	*P* value	OR(95%CI)	*P* value
Metabolic obesity phenotype		0.001		0.006
Metabolically healthy nonobesity (MHNO)	1.000(ref.)		1.000(ref.)	
Metabolically healthy obesity (MHO)	4.174 (1.468 - 11.868)	0.007	4.598 (1.476 -14.324)	0.009
Metabolically unhealthy nonobesity (MUNO)	1.469 (0.938 - 2.302)	0.093	1.390 (0.859 -2.250)	0.180
Metabolically unhealthy obesity (MUO)	3.608 (1.626 - 8.007)	0.002	3.296 (1.357 -8.007)	0.008
Uric acid(μmol/L)	1.001 (0.999 - 1.003)	0.368		
Total cholesterol (mmol/L)	1.036 (0.839 - 1.280)	0.740		
Low density lipoprotein cholesterol (LDL-c) (mmol/L)	1.263 (0.989 - 1.614)	0.061		
Albumin (g/L)	1.034 (1.012 - 1.056)	0.002	1.031 (0.990- 1.074)	0.135
Globulin(g/L)	1.051 (1.016 - 1.087)	0.004	1.017 (0.953 -1.085)	0.618
γ-Glutamyl Transferase (GGT) (μ/L)	0.998 (0.990 - 1.005)	0.530		
Alkaline phosphatase(U/L)	1.006 (1.002 - 1.011)	0.006	1.006 (1.001 -1.012)	0.015

### Excluding hypoglycemic individuals

In defining metabolic unhealthy status, hyperglycemia was considered one of the metabolic risk factors. We excluded participants with hypoglycemia (FPG <2.8 mmol/L) to account for the possibility that these individuals might have issues with their own blood glucose regulation. The results demonstrated that the risk of developing PCa in MUO individuals was 2.628 times higher than in MHNO individuals (OR= 2.628, 95%CI: 1.493- 4.626); **Table 7**.

**Table 7:** Exclusion of people with hypoglycemia.

Parameters	Univariate conditional logistic regression analysis		Multivariate conditional logistic regression analysis	
	OR(95%CI)	*P* value	OR(95%CI)	*P* value
Metabolic obesity phenotypes		0.008		0.010
Metabolically healthy nonobesity (MHNO)	1.000(ref.)		1.000(ref.)	
Metabolically healthy obesity (MHO)	1.397 (0.791 - 2.468)	0.249	1.410 (0.753 - 2.642)	0.283
metabolically unhealthy nonobesity (MUNO)	1.517 (0.899 - 2.562)	0.119	1.478 (0.838 - 2.608)	0.177
Metabolically unhealthy obesity (MUO)	2.493 (1.476 - 4.209)	<0.001	2.628 (1.493- 4.626)	0.001
Uric acid(μmol/L)	1.001 (0.999 - 1.003)	0.303		
Total cholesterol (mmol/L)	1.040 (0.836 - 1.295)	0.724		
Low density lipoprotein cholesterol (LDL-c) (mmol/L)	1.093 (0.858 - 1.392)	0.473		
Albumin (g/L)	1.042 (1.020 - 1.065)	<0.001	1.005 (0.967 -1.044)	0.816
Globulin(g/L)	1.084 (1.044 - 1.125)	<0.001	1.088 (1.021 - 1.161)	0.010
γ-Glutamyl Transferase (GGT) (μ/L)	0.994 (0.986 - 1.002)	0.136		
Alkaline phosphatase(U/L)	1.006 (1.001 - 1.011)	0.014	1.007 (1.001 - 1.012)	0.019

## Discussion

The combination of a metabolically unhealthy status and obesity (MUO), exhibited a higher risk of PCa compared to other combinations of metabolic status and BMI. Epidemiological evidence strongly supports the association between obesity and metabolic abnormalities (35, 36). This link is due to excessive nutrient intake, which alters several metabolic pathways involved in the pathogenesis of obesity-related diseases, including adipotoxicity, inflammatory pathways, aberrant adipokine expression, and adipose tissue senescence (37). Both metabolic status and obesity are strongly linked to inflammation (38), with chronic, low-grade inflammation playing a role in the pathogenesis of metabolic disorders (39). Obesity is characterized by the excessive accumulation of adipose tissue, which functions as a significant metabolic organ that secretes pro-and anti-inflammatory cytokines to modulate the inflammatory response (40). Increased proinflammatory cytokines in the adipose tissue of obese individuals reduce insulin sensitivity, leading to insulin resistance (41). Free fatty acids inhibit glucose transporter activity, reducing glucose uptake by muscle, and obesity can induce insulin resistance by increasing circulating free fatty acids (42). Gut leakage and microbiota dysbiosis in obese individuals result in elevated circulating levels of lipopolysaccharides, which are major contributors to adipose tissue inflammation and insulin resistance (43). Reduced extracellular matrix flexibility in adipose tissue is commonly associated with MUO (44). The expansion of adipose tissue is driven by the proliferation and hypertrophy of adipocytes and requires continuous remodeling of the extracellular matrix to accommodate this expansion (45). Adipose tissue fibrosis can lead to adipocyte apoptosis and chronic inflammation, characterized by infiltrating macrophages (46). Consequently, adipose tissue fibrosis with chronic inflammation is a hallmark of obesity-related metabolic disorders and a major cause of obesity-related metabolic dysfunction (47).

The risk of PCa was higher in both MHO and MUO individuals compared to MHNO individuals, suggesting that obesity plays a significant role in PCa. According to the World Health Organization (WHO), obesity results from a positive energy balance due to increased energy intake or decreased energy expenditure, leading to excessive fat accumulation in adipose tissue (48). Adipogenesis activation is a key marker of metabolic changes in tumor cells, and androgens, which are major regulators of PCa tumor cells, stimulate adipogenesis by interfering with the molecular mechanisms controlling cellular lipid homeostasis (49). Androgens cause a synergistic increase in the expression of several genes involved in triglyceride and cholesterol synthesis in various PCa cell lines. This effect is mediated by the androgen-induced activation of the secondary transcriptional regulator, sterol regulatory element-binding protein (SREBP). SREBP is crucial for intracellular lipid homeostasis and is involved in the synthesis of fatty acids and cholesterol (50). Elevated triglycerides, reduced HDL-c, and obesity are strongly associated with lower testosterone levels (51–54). The exact relationship between low testosterone and obesity or dyslipidemia is not fully understood. Some studies suggest that suppression of the hypothalamic-pituitary-gonadal (HPG) axis may be involved. Testosterone synthesis, regulated by the HPG axis, is reduced in the presence of obesity or dyslipidemia, and this reduction is linked to decreased insulin sensitivity and potential insulin resistance in HPG axis neurons (55). Visceral fat acts as an active endocrine tissue, and in patients with obesity or abnormal fat distribution, the secretion of adipose-specific cytokines (leptin, IL-6, TNF-α) is increased. These cytokines inhibit the secretion of gonadotropins and subsequently affect the HPG axis (56, 57). Low testosterone likely plays a role in regulating inflammation in various tissues, leading to increased levels of proinflammatory cytokines, which further inhibit testosterone release from the HPG axis (58). Previous studies have shown that obesity can lead to inflammation, and inflammation is attributed to overexpression of Monocyte Chemoattractant Protein-1 (MCP-1), which leads to defective insulin secretion, insulin resistance, and interference with other process of energy homeostasis. MCP-1 plays an important role in activating endothelial inflammatory markers, by stimulating IL-6 secretion and synthesis of Intercellular adhesion molecule-1 (ICAM-1) (59).

MUO individuals are at higher risk than MHO individuals, indicating that a metabolically unhealthy status increases the risk of developing PCa. Metabolically unhealthy patients exhibit higher levels of inflammation markers (IL-6, hs-CRP) and lymphocyte counts, and growing evidence suggests that inflammation influences PCa formation. Tumor-associated neutrophils, B cells, and complement components may promote PCa (60). The proportion of immune cell populations changes at different stages of PCa (61). Metabolic status affects HDL-c subclasses and is associated with insulin resistance and elevated triglycerides (62). Metabolic status changes and obesity may contribute to endothelial dysfunction. Metabolically unhealthy obesity (MUO) is associated with elevated biomarkers of endothelial dysfunction (63). One of the significant mechanisms for developing endothelial dysfunction in obese individuals is metabolic dysfunction, endothelial dysfunction is similarly one of the earliest vascular alterations observed in obesity as a result of obesity-related metabolic changes (64, 65). Hypertension, hyperglycemia, elevated triglycerides, and reduced high-density lipoprotein cholesterol (HDL-c) directly contribute to endothelial dysfunction (66). One study found that MHO might be a transient phenotype (36). A 20-year follow-up study revealed that about half of adults with MHO transitioned to MUO, only 10% of adults with MHO transitioned to MHNO, and adults with MHO were nearly eight times more likely to progress to MUO than adults with MHNO (67). Although MHO individuals are overweight, they still show a relatively beneficial hormonal and metabolic profile, with greater insulin sensitivity, a favorable immune profile, and reduced inflammation compared to the MUO population (18, 68).

This study has some limitations. Firstly, as a retrospective study, causal relationship between metabolic obesity phenotypes and PCa cannot be inferred, which limits the generalization of the results. The metabolic obesity phenotypes might change over time, indicating that physiological states at different time stages probably have an impact on the concentration of metabolic markers. Future prospective studies are needed to validate these findings. Secondly, we employ prostate puncture biopsy, and pathological tests to diagnose PCa, diagnostic methods are not sufficiently comprehensive. Finally, the criteria for measuring metabolically unhealthy status and obesity are not uniform and should be determined through further clinical trials. The advantage of this study is the use of propensity score matching, which reduces the effect of confounding factors, and the sample size calculation ensures sufficient certainty to detect statistical significance in the results.

## Conclusions

In conclusion, both MHO and MUO individuals are associated with a higher risk of developing PCa compared to MHNO individuals. Although the risk of PCa in MHO individuals is lower than in MUO individuals, this indicates that obesity is still a risk factor for PCa in metabolically healthy status. This emphasizes the importance of different types of metabolic obesity phenotypes in assessing PCa risk. Metabolically unhealthy status and obesity may be valid targets for PCa prevention.

## Data availability statement

The original contributions presented in the study are included in the article/**Supplementary Material**. Further inquiries can be directed to the corresponding authors.

## Ethics statement

The studies involving humans were approved by Ethics Committee of the First Affiliated Hospital of the Xinjiang Medical University (Approval No. 20220 308-166). The studies were conducted in accordance with the local legislation and institutional requirements. The participants provided their written informed consent to participate in this study. Written informed consent was obtained from the individual(s) for the publication of any potentially identifiable images or data included in this article.

## Author contributions

JW: Conceptualization, Formal analysis, Methodology, Software, Validation, Visualization, Writing – original draft. AA: Data curation, Formal analysis, Investigation, Writing – original draft. HQ: Data curation, Formal analysis, Software, Validation, Writing – original draft. NT: Conceptualization, Funding acquisition, Methodology, Supervision, Validation, Writing – review & editing. HA: Data curation, Funding acquisition, Methodology, Software, Supervision, Validation, Writing – review & editing.
