# Supplementary material for: Association between metabolic obesity phenotypes and the risk of developing prostate cancer: a propensity score matching study based on Xinjiang
Source: Front Endocrinol (Lausanne). 2024 Aug 6;15:1442740. doi: 10.3389/fendo.2024.1442740 (PMC11333236; doi:10.3389/fendo.2024.1442740)
Supplement: Supplementary file 2 [file DataSheet_2.pdf]

Supplementary Table 1 Integration of the lymphocytes percentage

Variable	*P*	OR (95%CI)
Metabolic obesity phenotype	0.014	
Metabolically healthy nonobesity (MHNO)		1.000(ref.)
Metabolically healthy obesity (MHO)	0.011	2.350(1.214-4.548)
Metabolically unhealthy nonobesity (MUNO)	0.095	1.727(0.910-3.278)
Metabolically unhealthy obesity (MUO)	0.002	2.651(1.417-4.958)
Albumin (g/L)	0.791	1.005(0.968-1.044)
Globulin(g/L)	0.055	1.062(0.999-1.130)
Alkaline phosphatase(U/L)	<0.001	1.010(1.005-1.016)
Lymphocytes percentage	0.044	1.025(1.001-1.050)

Supplementary Table 2 Integration of the eosinophils percentage

Variable	*P*	OR (95%CI)
Metabolic obesity phenotype	0.021	
Metabolically healthy nonobesity (MHNO)		1.000(ref.)
Metabolically healthy obesity (MHO)	0.017	2.219(1.152-4.273)
Metabolically unhealthy nonobesity (MUNO)	0.174	1.540(0.827-2.869)
Metabolically unhealthy obesity (MUO)	0.004	2.540(1.354-4.762)
Albumin (g/L)	0.934	1.002(0.965-1.040)
Globulin(g/L)	0.032	1.071(1.006-1.140)
Alkaline phosphatase(U/L)	<0.001	1.010(1.004-1.015)
Eosinophils percentage	0.030	1.121(1.011-1.244)
